# Tryptophan-rich domains of *Plasmodium falciparum* SURFIN_4.2_ and *Plasmodium vivax* PvSTP2 interact with membrane skeleton of red blood cell

**DOI:** 10.1186/s12936-017-1772-5

**Published:** 2017-03-20

**Authors:** Xiaotong Zhu, Yang He, Yifan Liang, Osamu Kaneko, Liwang Cui, Yaming Cao

**Affiliations:** 1 Department of Immunology, College of Basic Medical Science, China Medical University, Shenyang, 110122 Liaoning China; 2 98K 73B Seven-year Programme 127306, China Medical University, Shenyang, 110001 Liaoning China; 3 Department of Protozoology, Institute of Tropical Medicine (NEKKEN), Nagasaki University, 1-12-4 Sakamoto, Nagasaki, 852-8523 Japan; 4 Department of Entomology, The Pennsylvania State University, University Park, PA 16802 USA

**Keywords:** SURFIN, Tryptophan-rich domain, RBC membrane skeleton, F-actin, Spectrin

## Abstract

**Background:**

*Plasmodium falciparum* dramatically alters the morphology and properties of the infected red blood cells (iRBCs). A large group of exported proteins participate in these parasite-host interactions occurring at the iRBC membrane skeleton. SURFIN_4.2_ is one of iRBC surface protein that belongs to surface-associated interspersed protein (SURFIN) family. Although the intracellular tryptophan-rich domain (WRD) was proposed to be important for the translocation of SURFINs from Maurer’s clefts to iRBC surface, the molecular basis of this observation has yet to be defined. The WRDs of *P. falciparum* SURFIN proteins and their orthologous *Plasmodium vivax* subtelomeric transmembrane proteins (PvSTPs) show homology to the intracellular regions of PfEMP1 and Pf332, both of which are involved in RBC membrane skeleton interactions, and contribute to malaria pathology.

**Methods:**

Two transfected lines expressing recombinant SURFINs (NTC-GFP and NTC-4.2WRD2-GFP) of the 3D7 sequence were generated by transfection in *P. falciparum*. In vitro binding assays were performed by using recombinant WRDs of SURFIN_4.2_/PvSTP2 and inside-out vesicles (IOVs). The interactions between the recombinant WRDs of SURFIN_4.2_/PvSTP2 with actin and spectrin were evaluated by the actin spin down assay and an enzyme-linked immunosorbent assay based binding assays, respectively.

**Results:**

The recombinant SURFINs (NTC-4.2WRD2-GFP), in which the second WRD from SURFIN_4.2_ was added back to NTC-GFP, show diffused pattern of fluorescence in the iRBC cytosol. Furthermore, WRDs of SURFIN_4.2_/PvSTP2 were found to directly interact with the IOVs of RBC, with binding affinities ranging from 0.26 to 0.68 μM, values that are comparable to other reported parasite proteins that bind to the RBC membrane skeleton. Further experiments revealed that the second WRD of SURFIN_4.2_ bound to F-actin (*K*_d_ = 5.16 μM) and spectrin (*K*_d_ = 0.51 μM).

**Conclusions:**

Because PfEMP1 and Pf332 also bind to actin and/or spectrin, the authors propose that the interaction between WRD and RBC membrane skeleton might be a common feature of WRD-containing proteins and may be important for the translocation of these proteins from Maurer’s clefts to the iRBC surface. The findings suggest a conserved mechanism of host-parasite interactions and targeting this interaction may disrupt the iRBC surface exposure of *Plasmodium* virulence-related proteins.

**Electronic supplementary material:**

The online version of this article (doi:10.1186/s12936-017-1772-5) contains supplementary material, which is available to authorized users.

## Background

In spite of extensive control efforts, malaria continues to be a major health problem worldwide, causing approximately 438,000 deaths in 2015. In Africa alone, the death toll reached 292,000 among children under five years old [1]. The malignant tertian parasite *Plasmodium falciparum* accounts for the majority of fatal malaria infections. Severe pathologies such as organ failure and dysfunction, cerebral malaria, and placental malaria are most often associated with sequestration of the infected red blood cells (iRBCs) into the deep microcapillaries of these organs by adhering to endothelial cells. Cytoadherence is mediated by parasite proteins exported to the iRBC membrane. These proteins are first transported across the parasite plasma membrane and the parasitophorous vacuole membrane (PVM). Then they are sorted and translocated through the Maurer’s clefts and finally inserted into the iRBC membrane [2, 3]. Maurer’s clefts are membranous structures involved in sorting and translocating parasite proteins to the iRBC membrane [4, 5]. These extensive modifications of the iRBC dramatically alter its morphology, antigenicity and functions, including the appearance of knob protrusions on the iRBC surface, increased rigidity and poor deformability of iRBC, and increased adhesiveness of the iRBC to the endothelium [4, 6, 7]. Some exported proteins such as PfEMP1, PfEMP3, MESA, Pf332, PfSBP1, KAHRP1, and RESA interact with RBC membrane skeleton [7–13]. In addition, PfEMP1 family proteins encoded by the *var* gene family bind to host factors such as CD36, ICAM-I, and CSA, mediating cytoadherence of the iRBCs and leading to severe pathologies.

Among *P. falciparum* proteins that contain tryptophan-rich residues are the SURFIN family proteins. SURFIN_4.2_ is one of the iRBC-exported proteins and is encoded by a small family of surface-associated interspersed (*surf*) genes consisting of 10 members in the *P. falciparum* genome [14]. *Plasmodium falciparum* SURFINs form one clade with the *Plasmodium vivax* subtelomeric transmembrane proteins (PvSTPs) [15]. The intracellular tryptophan-rich domains (WRDs) of SURFIN/PvSTP are related to the sequences of the intracellular regions of PfEMP1 and Pf332 [14]. SURFIN_4.2_ localizes to Maurer’s clefts and has been reported to be trafficked to the surface of the iRBC together with RIFIN and PfEMP1 [14]. Thus, SURFIN/PvSTP proteins are potential immune targets and malaria vaccine candidates [16, 17]. For another member SURFIN_4.1_, the N-terminal 50 amino acids, transmembrane domain, and adjacent intracellular region contain sufficient information for recruiting a recombinant protein into the classical ER/Golgi secretory pathway, and for efficient translocation across the PVM to the Maurer’s clefts [18]. The mechanism by which SURFIN proteins are anchored into the iRBC membrane has yet to be elucidated, but recombinant SURFIN_4.2_ possessing the intracellular WRD can be cleaved by surface treatment of iRBC with proteinase K, suggesting the WRD of SURFIN_4.2_ may be responsible for transport of the protein from Maurer’s clefts to the iRBC membrane [19]. Interestingly, intracellular region of Pf332 that is homologous to the SURFIN WRD is found to associate with actin filaments of RBC membrane skeleton [12]. In the case of PfEMP1, the intracellular VARC region (also known as the acidic terminal sequence, ATS) having homology with WRD binds to host spectrin-actin [4, 20, 21]. Thus, this study aimed to identify host RBC proteins that may associate with SURFIN_4.2_ WRD. This could provide an important insight into the molecular basis of trafficking of SURFIN proteins from Maurer’s cleft to iRBC surface. This study revealed binding of WRDs of SURFIN_4.2_ and PvSTP2 to RBC membrane skeleton proteins, and interactions between the second WRD of SURFIN_4.2_ with actin and spectrin.

## Methods

### Construction of plasmids for *Plasmodium falciparum* transfection

Plasmids used to transfect *P. falciparum* were prepared based on the Multisite Gateway System (Thermo Scientific, USA). The template plasmids pENT12-SURFIN_4.1_^2Myc-N-T-Cyt^ and pENT12-SURFIN_4.1_^2Myc-N-T-Cyt-*Stu*I^ were generated using the Q5^®^ Site-Directed Mutagenesis Kit (NEB) [22] based on the initially generated pENT12-SURFIN_4.1_^N-T-Cyt^ plasmids (Primer list; Additional file 1) [18]. For *P. falciparum* transfection, the region encoding WRD2 of SURFIN_4.2_ was amplified by PCR with primers listed in Additional file 1. PCR fragments were analysed by electrophoresis on a 1.2% agarose gels, and purified by using TaKaRa MiniBEST DNA Fragment Purification Kit Ver.4.0 (Takara, Japan). The purified PCR product was ligated into the *Stu*I site of pENT12-SURFIN_4.1_^2Myc-N-T-Cyt-*Stu*I^ plasmid to generate the pENT12-SURFIN_4.1_^2Myc-N-T-Cyt-4.2WRD2^ plasmid. All constructs were verified by restriction digestion and sequencing. Ultimately, pENT12-SURFIN_4.1_^2Myc-N-T-Cyt^ and pENT12-SURFIN_4.1_^2Myc-N-T-Cyt-4.2WRD2^ plasmids were recombined with the destination plasmid pCHD43-II (modified based on pCHD-3/4 plasmid [23]) with pENT41-pfHsp86-5′UTR and pENT23-GFP_m2_ using the Gateway Multisite LR recombination reaction according to the manufacturer’s instruction.

### Preparation of recombinant proteins

For recombinant protein expression in *E. coli*, specific primers (Additional file 1) were designed with reference to the *surf*_*4.2*_ and *pvstp2* nucleotide sequences and used to PCR amplify DNA fragments encoding WRDs of SURFIN_4.2_ and Pf332 from *P. falciparum* 3D7 [amino acid positions: SURFIN_4.2_ WRD1 (959–1201); SURFIN_4.2_ WRD2 (1349–1567); SURFIN_4.2_ WRD2-1 (1349–1499); SURFIN_4.2_ WRD3 (1729–1990); SURFIN_4.2_ CRD (1–197); Pf332 WRD (5565–5825)] and WRD of PvSTP2 from *P. vivax* Sal-I cDNA (amino acids 592–825). Approximately 2 mg recombinant SURFIN_4.2_WRD2 (amino acids 1349–1567) tagged with His tag expressed in a yeast expression system were obtained from the Gene Create Company (Wuhan, China). A DNA fragment encoding a region of KAHRP protein (amino acid positions 320–451), which contain 72 amino acid spectrin-binding fragments (amino acid positions 370–441, [24]) was also amplified from 3D7 cDNA as a positive control for spectrin binding assays (primers listed in Additional file 1). For *surf*_*4.2*_, *pvstp2*, and *kahrp* ^320–451^, PCR products were cloned into the pBADR-DEST49 vector using the Gateway cloning technology (Thermo Scientific, USA). The inserts were verified by sequencing and plasmids were designated as pBADR-SURFIN_4.2_^WRD1^ (SURFIN_4.2_^WRD1^), pBADR-SURFIN_4.2_^WRD2^ (SURFIN_4.2_^WRD2^), pBADR-SURFIN_4.2_^WRD2-1^ (SURFIN_4.2_^WRD2-1^), pBADR-SURFIN_4.2_^WRD3^ (SURFIN_4.2_^WRD3^), pBADR-SURFIN_4.2_^CRD^ (SURFIN_4.2_^CRD^), pBADR-PvSTP2^WRD^ (PvSTP2^WRD^), and pBADR-KAHRP^320–451^ (KAHRP^320–451^). For Pf332 (positive control), the PCR product was cloned into pET-32a (+) to generate pET32a-Pf332^WRD^ (Pf332^WRD^). Recombinant WRDs were expressed in *E. coli* BL21 Rosetta-gamiB (DE3) after induction with 0.001% l-arabinose or 1 mM isopropyl β-d-1-thiogalactopyranoside at 20 °C for 16 h. The bacterial cells were then collected by centrifugation (8000×*g* for 10 min, 4 °C), resuspended, and lysed by BugBuster Master Mix (Merck). After 20 cycles of sonication (10 s pulses with 3 s intervals between each cycle), the lysates were collected by centrifugation (11,000×*g*, 4 °C) and supernatants were purified on -IDA-Sefinose TM Resin (Sangon Biotech, China) according to the manufacturer’s protocol. The recombinant WRDs were dialyzed against phosphate buffed saline (PBS) at 4 °C for 72 h with buffer changing every 24 h, and concentrated by using Amicon^®^ Ultra-0.5 (Millipore, USA). The concentrations of recombinant proteins were determined by using the TaKaRa BCA Protein Assay Kit (Takara, Japan). The molecular weight (MW) of each recombinant WRDs were estimated using ExPASy Bioinformatics Resource Portal (http://ca.expasy.org/tools/pi_tool.html [25]). All His-tagged recombinant proteins were clarified by ultracentrifugation at 150,000×*g* for 30 min before use.

### Parasite culture and transfection

The *P. falciparum* 3D7 line was cultured in vitro in RPMI medium supplemented with 5% human serum plus 0.25% Albumax I according to the standard method as previously described [26]. Transfection was performed as described [18] and parasites were selected with WR99210 (a gift from D. Jacobus, Jacobus Pharmaceutical Co. Inc., USA) first at a concentration of 5 nM and then at 10 nM when parasites reappeared.

### Indirect immunofluorescence assay (IFA)

Thin smears of *P. falciparum*-iRBCs on glass slides were briefly dried and fixed with 4% paraformaldehyde and 0.005% glutaraldehyde in PBS for 15 min at room temperature. After rinsing with 50 mM glycine, the slides were blocked with 5% of skim milk in PBS for 30 min at 37 °C. The slides were first incubated with mouse monoclonal anti-GFP (Roche, Switzerland) and rabbit anti-EXP2 antiserum at 1:1000 dilutions or rabbit anti-SBP1 at 1:500 dilutions or rat anti-PfEMP1 1:500 dilutions at 37 °C for 1 h. Then, the slides were incubated with Alexa-Fluor 488-conjugated goat anti-mouse or Alexa-Fluor 594-conjugated goat anti-rabbit or Alexa-Fluor 594-conjugated goat anti-rat antibodies at 1:2000 (Thermo Scientific, USA) at 37 °C for 30 min. DAPI (Sigma) was used at 1 μg/ml as a counterstain of parasite nuclei. ProLong^®^ Diamond Antifade Mountant (Thermo Scientific, USA) was applied onto the slide to reduce quenching under the UV light. The slides were viewed with a Nikon ECLIPSE 80i microscope. The signal intensity of immunofluorescence in *P. falciparum* transfectants was measured with ImageJ software (1.44p; http://rsbweb.nih.gov/ij/).

### In vitro binding assays

Inside-out-vesicles (IOVs) of normal human RBCs were prepared using a previously described method [12]. In vitro binding assays using IOVs were conducted by an enzyme-linked immunosorbent assay (ELISA) format. Briefly, prepared IOVs diluted in an incubation buffer (IB; 138 mM NaCl, 5 mM KCl, 6 mM Na_2_HPO_4_, 5 mM glucose, pH 9.0) were coated onto 96-well plates (Dynatech Laboratories Inc., USA) overnight at 4 °C. The plate was washed then blocked with 5% (w/v) bovine serum albumin (BSA) in PBS for 1 h at room temperature. Serially diluted His-tagged recombinant WRD proteins, including Pf332^WRD^, SURFIN_4.2_^WRD1^, SURFIN_4.2_^WRD2^, SURFIN_4.2_^WRD3^, and PvSTP2^WRD^ (0.25–10 μM) were added to the IOVs-coated plates and incubated overnight at 4 °C. His-tagged protein from the empty vector pET-32a (+) was used as a negative control (detailed protein sequence information of His-tagged unrelated protein is shown in Additional file 2). Plates were washed five times, followed by the detection of the recombinant proteins with the HRP-conjugated anti-His tag antibody (Abcam, USA). Colour development was done by adding 100 μl of TMB microwell peroxidase substrate. After reaction at room temperature for 5 min, 50 μl of 2 mM H_2_SO_4_ were added to each well to terminate the reaction, and absorbance at 450 nm was measured using an ELISA microplate reader. For internal control, BSA was used instead of IOVs. A saturation of binding curve was constructed from the optical density (OD) values of the representative binding assay after subtraction of the signal obtained from the BSA control. The dissociation constant (*K*_d_) was determined by regression analysis of the binding curves.

### Actin co-sedimentation assays

The specificity and affinity of the interaction between SURFIN_4.2_^WRD2^-His and F-actin were detected by using the Actin Binding Protein Spin-Down Assay kit (Cytoskeleton, USA). Briefly, 250 µl of G-actin (Cytoskeleton, USA) at 1 mg/ml was polymerized to F-actin by adding 25 µl of actin polymerization buffer (500 mM KCl, 20 mM MgCl_2_, and 10 mM ATP) into the G-actin solution for 1 h at room temperature. F-actin (7 μM) was incubated with SURFIN_4.2_^WRD2^-His protein (titrated from 12.5 to 0.0 μM), the His-tagged protein from the empty vector as a negative control (7.0 μM), and the positive control Pf332^WRD^ (4.2 μM) at room temperature for 30 min, followed by ultracentrifugation at 150,000×*g* for 1.5 h at 24 °C using an ultracentrifuge (Himac CS150GXL; Hitachi, Japan). The resulting pellet was resuspended in 50 μl of 1× Pierce™ Lane Marker Reducing Sample Buffer (Thermo Scientific, USA) to the original sample volume, resolved by 10% SDS-PAGE, transferred to PVDF for Western blot detection using either HRP-conjugated anti-6× His tag antibody, rabbit anti-actin antibody (Sigma, China), or mouse anti-actin antibody (clone 1A4, Thermo Scientific, USA). The Western blot was analysed by densitometry using the ImageJ software. After subtracting the nonspecific binding, a binding curve using the densitometric data was plotted and the dissociation constant (*K*_d_) and *B*_max_ were determined by nonlinear regression analysis (one-site specific binding model) using GraphPad Prism 6 software. The specificity of interaction between SURFIN_4.2_^WRD2-1^-His, SURFIN_4.2_^CRD^-His, and SURFIN_4.2_^WRD2^-His and F-actin was also evaluated by using the actin spin down assay following the same protocol.

### In vitro binding assay with spectrin

His-tagged recombinant SURFIN_4.2_^WRD2^ was diluted in PBS and binding assays were performed in a similar manner to the IOV binding assays. Briefly, 100 ng of purified human spectrin (Sigma) was coated on a 96-well plate at 4 °C overnight. After washing the plate and blocking with 5% BSA at room temperature for 1 h, actin (6 µM), BSA (6 µM), and recombinant SURFIN_4.2_^WRD2^-His (6 µM) were added to the spectrin-coated plate and incubated overnight at 4 °C. After washing with PBS three times, the bound proteins were stripped off from the plates with a SDS sample buffer, and analysed by Western blot using a monoclonal mouse 6× His tag antibody (Thermo Scientific, USA), rabbit anti-actin antibody. Spectrin dimer and BSA were evaluated by Coomassie Brilliant Blue staining. In a parallel study, the bound proteins, including KAHRP^320−451^ (positive control), His (negative control), and recombinant SURFIN_4.2_^WRD2^-His were processed for quantification using an ELISA-based in vitro binding format as described above.

## Results

### Effect of the SURFIN_4.2_ WRD2 on the recombinant mini-SURFIN protein localization in *P. falciparum*-iRBC

**Fig. 1:**
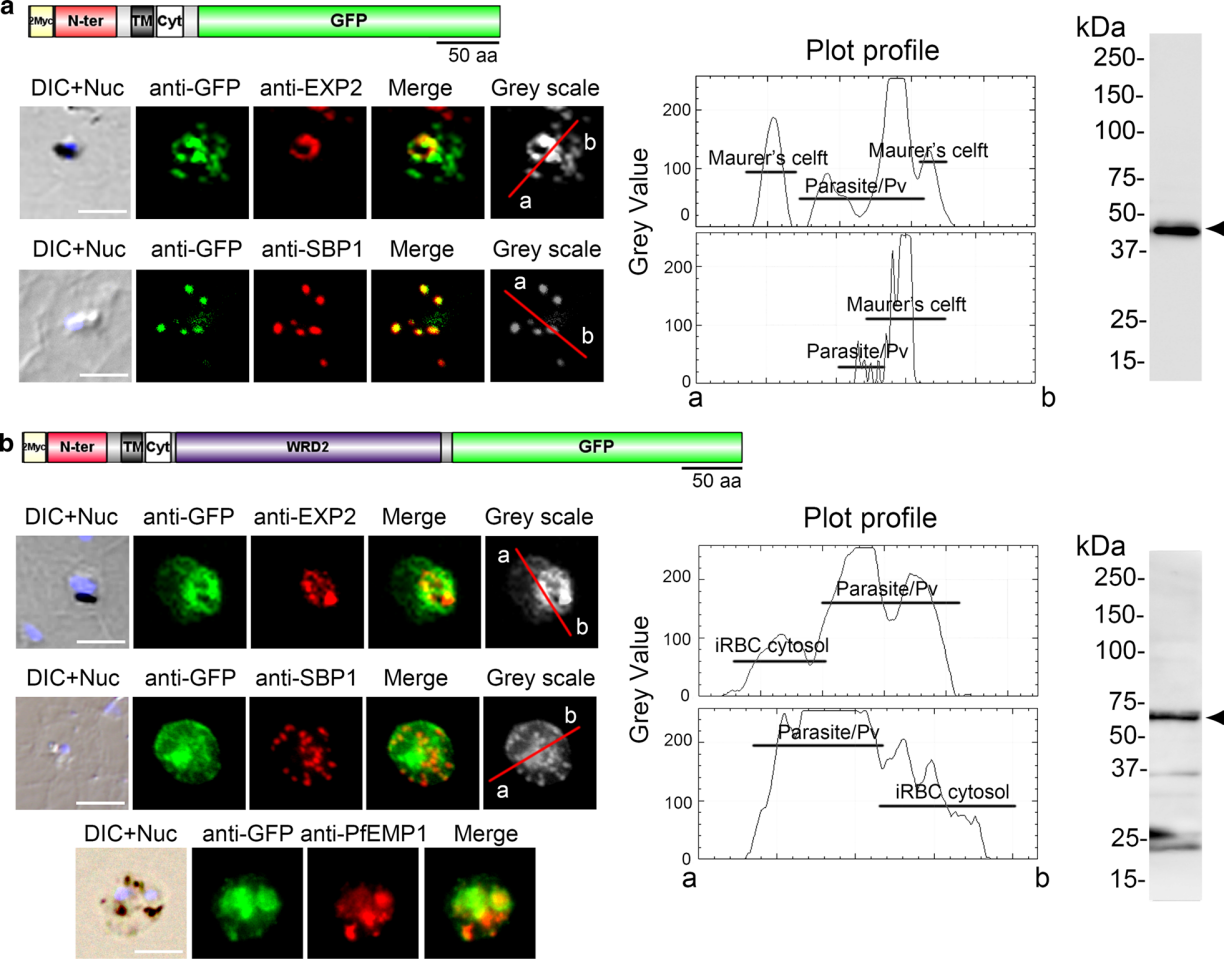
Localization and signal intensity comparison of mini-SURFIN_4.1_, NTC-GFP and NTC-4.2WRD2-GFP, in *P. falciparum*-iRBC. **a** Co-localization of NTC-GFP with the PVM marker EXP2 and Maurer’s cleft marker SBP1. *Upper panel* Schematic drawing of NTC-GFP. *Lower panels* Representative fluorescence images showing the co-localization of NTC-GFP with EXP2 and SBP1. **b** Co-localization of NTC-4.2WRD2-GFP with EXP2, SBP1 and PfEMP1. *Upper panel* Schematic drawing of NTC-4.2WRD2-GFP. *Lower panels* Representative fluorescence images showing the co-localization of NTC-4.2WRD2-GFP with EXP2, SBP1 and PfEMP1. The differential interference contrast images merged with nucleus stained with DAPI (DIC + Nuc), fluorescence images with mouse anti-GFP antibody (anti-GFP), PVM location with rabbit anti-EXP2 antibody (anti-EXP2) or Maurer’s cleft location with rabbit anti-SBP1 antibody (anti-SBP1), or rat anti-PfEMP1 antibody (anti-PfEMP1), and merged image are shown. *Bar* 5 μm. Scale refers to the residue number of recombinant SURFIN_4.1_ protein. Plot profiles of signal intensities evaluated by ImageJ software are shown by a *grey* scale on the *right side* of each immunofluorescence panel, along with the Western blot data for recombinant SURFINs. The bands at the predicted size for the recombinant NTC-GFP and NTC-4.2WRD2-GFP are marked with *arrowheads*. Parasite/PV indicates parasite cytosol or parasitophorous vacuole

The intracellular regions of PfEMP1 and Pf332 are known to interact with the RBC membrane skeleton components, including actin and spectrin. The alignment between WRDs of SURFIN_4.2_/PvSTP2 and intracellular regions of PfEMP1 and Pf332 show positionally conserved amino acids in RBC membrane skeleton binding regions (Additional file 3). Furthermore, based on the fact that the mini-SURFIN_4.1_ consisting of the N-terminus, the transmembrane region, and a short cytoplasmic tail of SURFIN_4.1_ fused with GFP at C-terminus was able to be trafficked to Maurer’s clefts, two plasmids were generated expressing mini-SURFIN_4.1_ fused with or without SURFIN_4.2_ WRD2, which share high similarity between SURFIN_4.1_ and SURFIN_4.2_ proteins (NTC-4.2WRD2-GFP or NTC-GFP, respectively; Additional file 4). IFA with the PVM marker EXP2 revealed that NTC-GFP was exported beyond the PVM into infected RBC (iRBC) cytosol, and the signals showed a dotted pattern, which co-localized with the Maurer’s cleft marker SBP1, suggesting Maurer’s cleft localization (Fig. 1a). The dominant fluorescence signals were detected in parasite cytosol and Maurer’s cleft, as shown in plot profiles by Image J analysis. However, NTC-4.2WRD2-GFP produced signals beyond the PVM with a diffused localization pattern in the iRBCs, which only partially co-localized with the Maurer’s cleft marker SBP1 and with PfEMP1, suggesting that it was likely transported beyond Maurer’s clefts and to the RBC cytosol or membrane (Fig. 1b), a result that is consistent with previous reports [6, 19].

### Recombinant WRD2 of SURFIN_4.2_ is associated with the RBC IOVs

**Fig. 2:**
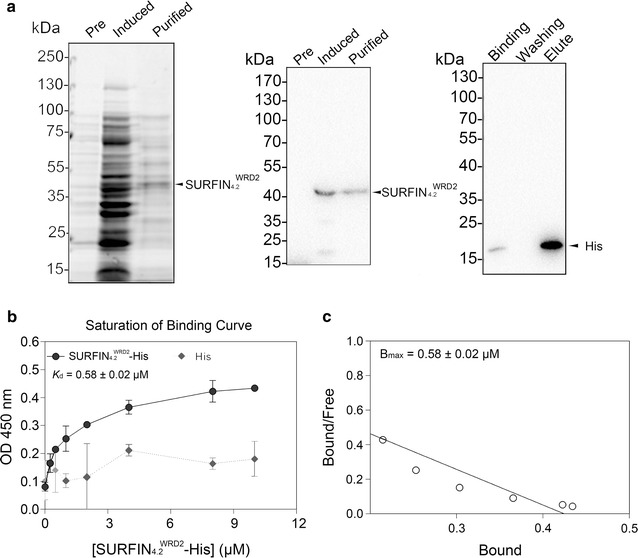
Binding assays with inside-out vesicles (IOVs). **a** Expression and purification of recombinant SURFIN_4.2_^WRD2^-His. The Coomassie Brilliant Blue staining (*left*) and Western blot analysis (*middle*) of the total *E. coli* lysates of the uninduced (Pre), induced, and purified culture of recombinant SURFIN_4.2_^WRD2^-His (calculated MW, 46.5 kDa). *Right panel* shows a Western blot for the bound, washed, and eluted fractions of the His tag alone (20.4 kDa). The bands at the predicted size for the recombinant protein were marked with *arrowheads*. **b** A saturation binding curve using ELISA-based protein interaction analysis. The dissociation constant (*K*_d_) was determined to be 0.58 ± 0.02 μM. **c** Scatchard plot of SURFIN_4.2_^WRD2^-His and IOVs interaction. *B*_max_ = 0.44 ± 0.03 μM

To evaluate the interaction of WRD2 of SURFIN_4.2_ with RBC membrane skeleton, binding assays were performed using the recombinant His-tagged SURFIN_4.2_ WRD2 protein (SURFIN_4.2_^WRD2^-His) and IOVs prepared from normal human RBC (Fig. 2a). A His-tagged unrelated protein (His-tag control protein) was used as a negative control. SDS-PAGE of the IOVs followed by Coomassie Brilliant Blue staining confirmed the presence of the major RBC membrane skeleton proteins, including spectrin, protein 4.1 and actin (Additional file 5). SURFIN_4.2_^WRD2^-His bound to the IOVs in a dose-dependent manner and saturated at ~10 µM of SURFIN_4.2_^WRD2^-His, whereas only a trace level of His-tag control protein was detected under identical conditions (Fig. 2b), suggesting that the binding between SURFIN_4.2_^WRD2^-His and IOVs was specific. To characterize the affinities of SURFIN_4.2_^WRD2^-His with IOVs, Scatchard analysis was performed. The results showed that the *K*_d_ and *B*_max_ values of the affinities of SURFIN_4.2_^WRD2^-His to IOV were 0.58 ± 0.02 µM and 0.44 ± 0.03 µM, respectively (Fig. 2c). Together, these interaction assays suggest that the WRD2 of SURFIN_4.2_ binds to the RBC membrane skeleton.

### Other WRDs of *P. falciparum* SURFIN_4.2_ and *P. vivax* PvSTP2 also bind to the RBC IOVs

**Fig. 3:**
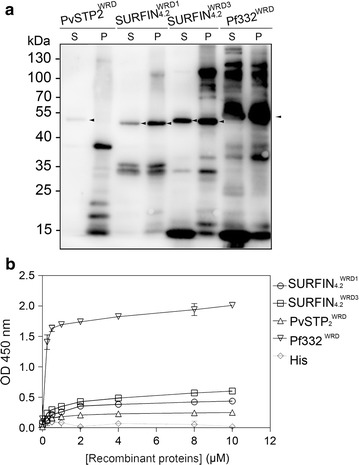
Interactions of recombinant WRDs of SURFIN_4.2_ and PvSTP2 with IOVs. **a** His-tagged recombinant WRD proteins were purified using the Ni-IDA-Sefinose TM Resin and analysed by Western blot. *S* Soluble fraction, *P* pellet fraction. *Arrowheads* indicate bands of His-tagged recombinant PvSTP2^WRD^ (calculated MW, 46.5 kDa), SURFIN_4.2_^WRD1^ (48.9 kDa), SURFIN_4.2_^WRD3^ (51.0 kDa), and Pf332^WRD^ (46.5 kDa). Soluble fractions of each recombinant protein were used for the in vitro interaction assays with membrane skeleton of RBC. **b** Saturation curves of the binding of recombinant WRD proteins to IOVs were measured by ELISA-based protein interaction analysis

Potential interactions ofWRD1 and WRD3 of SURFIN_4.2_ and WRD of PvSTP2, a *P. vivax* SURFIN-ortholog, with the RBC IOVs were further examined. The purified recombinant SURFIN_4.2_^WRD1^-His, SURFIN_4.2_^WRD3^-His, PvSTP2^WRD^-His, and Pf332^WRD^-His (positive control) proteins were examined by the IOV interaction assays (Fig. 3a). ELISA and Scatchard analyses showed that the SURFIN_4.2_^WRD1^-His, SURFIN_4.2_^WRD3^-His, and PvSTP2^WRD^-His all bound to the RBC IOVs in a dose-dependent manner (Fig. 3b; Additional file 6) with the *K*_d_ and *B*_max_ values of the interactions being 0.68 ± 0.16 and 0.46 ± 0.00, 0.53 ± 0.10 and 0.59 ± 0.03, and 0.26 ± 0.03 and 0.25 ± 0.01 µM, respectively. *K*_d_ and *B*_max_ values of positive control Pf332^WRD^-His were 0.10 ± 0.02 and 1.92 ± 0.04 µM, respectively, and those of negative control were −0.01 ± 0.20 and 0.22 ± 0.04 µM, respectively.

### Interaction of His-tagged recombinant SURFIN_4.2_^WRD2^ with F-actin

**Fig. 4:**
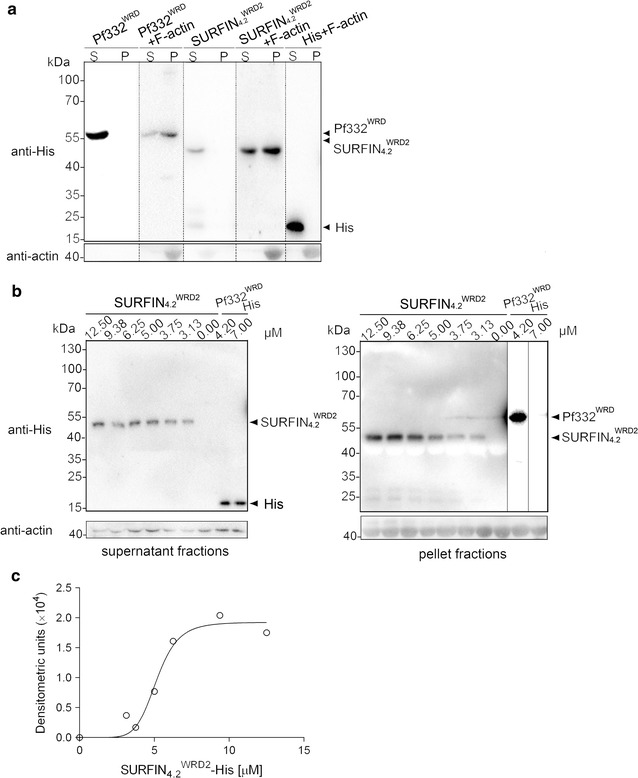
Binding of the His-tagged recombinant SURFIN_4.2_^WRD2^ to F-actin. **a** The supernatant and pellet fractions of SURFIN_4.2_^WRD2^-His (SURFIN_4.2_^WRD2^), Pf332^WRD^-His (Pf332^WRD^), and His-tag control protein after F-actin co-sedimentation assay were analysed by Western blot with anti-His tag or anti-actin antibodies. *Arrows* indicate recombinant protein bands with the expected size. *S* Supernatant fraction, *P* pellet fraction. **b** Western blot of the supernatant (*left*) and pellet (*right*) fractions after F-actin co-sedimentation with serially diluted SURFIN_4.2_^WRD2^-His, control His-tagged control protein, and Pf332^WRD^-His are shown. Because the signal of the Pf332^WRD^ detected in the pellet fraction was saturated, the intensity of this lane was reduced. **c** Saturation curves of the binding of SURFIN_4.2_^WRD2^-His to F-actin based on the F-actin co-sedimentation assays

To determine whether actin in the RBC membrane skeleton is responsible for the binding to the recombinant SURFIN_4.2_^WRD2^-His, a polymerized F-actin co-sedimentation assay was carried out. SURFIN_4.2_^WRD2^-His was detected in the pellet fraction, whereas His tag alone was not co-precipitated, indicating that the SURFIN_4.2_^WRD2^-His binding to F-actin was specific (Fig. 4a). These results were further proved by using SURFIN_4.2_^WRD2^-His expressed in a yeast expression system in the actin spin down assay, where the majority of SURFIN_4.2_^WRD2^-His was detected in the pellet fraction (Additional file 7). However, the recombinant protein that contains a shortened fragment of SURFIN_4.2_ WRD2 region, SURFIN_4.2_^WRD2-1^-His, was not enriched in the pellet, indicating that essential F-actin binding motifs may exist in the WRD2 region of SURFIN_4.2_ (Additional file 7). Interestingly, testing the cysteine-rich domain (CRD) of SURFIN_4.2_, originally selected as a negative control, also detected interaction between SURFIN_4.2_^CRD^-His with F-actin (Additional file 7). The binding of the positive control Pf332^WRD^-His to F-actin was also confirmed (Fig. 4a). The binding affinity of SURFIN_4.2_^WRD2^-His to F-actin was determined by incubating F-actin with Pf332^WRD^-His, His-tag control protein, or serially diluted SURFIN_4.2_^WRD2^-His. Western blot of a representative experiment for both the supernatant and pellet fractions is shown in Fig. 4b. Nonlinear regression analysis showed that the binding of SURFIN_4.2_^WRD2^-His to F-actin was specific and saturable. Notably, the *K*_d_ and *B*_max_ values of SURFIN_4.2_^WRD2^-His binding to F-actin were 5.16 ± 0.29 and 1.92 ± 0.15 μM, respectively (Fig. 4c). These data indicated that SURFIN_4.2_^WRD2^-His was able to bind to F-actin.

### His-tagged recombinant SURFIN_4.2_^WRD2^ binds to spectrin

**Fig. 5:**
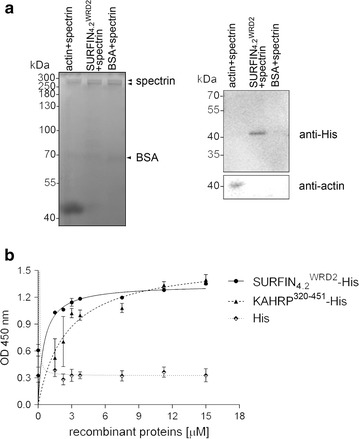
Binding of SURFIN_4.2_^WRD2^-His to spectrin. **a** Immunoblots from the spectrin binding assay. F-actin (positive control), SURFIN_4.2_^WRD2^-His, and BSA (negative control) were stripped from spectrin-coated wells, separated by SDS-PAGE, and stained by Coomassie Brilliant Blue (*left panel*). Immunoblot was performed with anti-His tag antibody and anti-actin antibody (*right panel*). Expected sizes of SURFIN_4.2_^WRD2^-His, actin, spectrin dimer, and BSA were ~46.5, 43, 250, and 66.4 kDa, respectively (indicated by the *arrowheads*). **b** The binding affinity of SURFIN_4.2_^WRD2^-His, KAHRP^320–451^-His, and His protein with spectrin. A binding saturation curve was constructed based on values obtained by the ELISA-based assay using serially diluted SURFIN_4.2_^WRD2^-His and KAHRP^320–451^-His. An unrelated His protein was used as the negative control

Finally, the study examined whether the recombinant SURFIN_4.2_^WRD2^-His interacts with another component of the RBC skeleton, spectrin. An in vitro binding assay with recombinant human spectrin followed by Western blot analysis detected the positive control actin and SURFIN_4.2_^WRD2^-His from the spectrin-bound fraction, whereas only a residual level of BSA (as the negative control) was detected (Fig. 5a). Scatchard analysis of the ELISA-based spectrin-binding assay of SURFIN_4.2_^WRD2^-His by using the His-tagged KAHRP spectrin-binding fragment as a positive control (amino acids 320–451, KAHRP^320–451^-His; Additional file 8) and His only protein as a negative control revealed that the *K*_d_ and *B*_max_ values of SURFIN_4.2_^WRD2^-His and spectrin interaction were 0.51 ± 0.38 and 1.34 ± 0.13 µM, respectively (Fig. 5b). Taken together, these data indicate that the WRD2 of SURFIN_4.2_ was able to bind to spectrin with modest affinity.

## Discussion

The human RBCs are enucleated, terminally differentiated cells, and packed with hemoglobin that is responsible for oxygen and carbon dioxide transportation in the circulatory system. RBCs contain a membrane bound skeleton network primarily comprised of spectrin, actin, and protein 4.1, which make the RBCs highly deformable to pass through the capillaries/reticuloendothelial system without fragmentation. However, after malaria parasite invasion, the morphology and functions of iRBCs are dramatically modified, a process that contributes to the disease pathology. Malaria parasites as ‘master renovators’ of their host cells achieve these modifications by exporting hundreds of proteins into the iRBC cytosol [27]. Some of these exported proteins are located to the surface of iRBC, such as the cytoadhesins PfEMP1, STEVOR, and RIFIN, and others including RESA, MESA, and PfEMP3, which become associated with the RBC membrane skeleton [7, 10, 13, 28–31].

Furthermore, some proteins reside in the Maurer’s cleft, which acts as a sorting depot for proteins *en route* to the surface of iRBC, including SBP1, REX1, and MAHRP1, which are responsible for PfEMP1 to be inserted into the plasma membrane of iRBC [10, 32, 33]. Extensive studies in protein trafficking have identified the *Plasmodium* exported element (PEXEL) in numerous parasite exported proteins. However, an even probably larger group of *Plasmodium* parasites also exported is PEXEL-negative proteins (PNEPs), including PfEMP1, SURFIN family, and some Maurer’s cleft dotting proteins, which typically contain an internal transmembrane domain that functions as an ER sorting signal, and an essential N-terminal signal responsible for further localization to the iRBC surface or remaining in the cytosol to help remodeling the iRBCs [18, 34–37]. The trafficking mechanism of most of the PNEPs including SURFIN is not fully understood.

As one of the variant surface antigens (VSAs), *surfin/pvstp* gene family possesses 10 members in *P. falciparum* and two members in *P. vivax*; some were also detected in the genomes of *Plasmodium ovale curtisi* and *Plasmodium ovale wallikeri* [14, 16, 38, 39]. Furthermore, previous hierarchical clustering analysis identified two *surfin/pvstp* genes in *Plasmodium gallinaceum* (*PgSurf1* and *PgSurf2*), indicating that SURFINs are also conserved outside the human malaria parasites [40]. Multiple sequence alignment revealed that SURFIN/PvSTP proteins, *P. falciparum* PfEMP1 and Pf332, and *Plasmodium knowlesi* SICA*var* are interrelated through a modular and structurally conserved intracellular WRD [14, 40]. PfEMP1 WRD binds to host spectrin-actin with high affinity (*K*_d_ = 0.04 µM), and also interacts electrostatically with four linear sequence motifs in KAHRP to form a fuzzy complex and to govern the phenomena of knob formation and cytoadherence of iRBC [4, 20, 21]. Furthermore, a 260-residue sequence within the WRD of Pf332 specifically interacts with F-actin (*K*_d_ = 0.60 μM, [12]). In the current study, WRD2 shared the most similarity between two known exported SURFINs, SURFIN_4.1_ and SURFIN_4.2_. Previous data have shown that recombinant SURFIN_4.2_ containing WRDs were mainly detected in Triton X-100 insoluble fractions, compared to the one without WRD, and also exhibited a unique localization pattern in the iRBC cytosol, implying a direct interaction of these SURFIN_4.2_ with the RBC membrane [6]. Consistently, by fusing the conserved WRD2 of SURFIN_4.2_ with the minimum Maurer’s cleft targeting motifs present in SURFIN_4.1_ [18], the recombinant NTC-4.2WRD2 also was targeted to the iRBC cytosol. Sequence conservation among WRDs suggested that WRD serves as a domain responsible for the binding to RBC membrane skeleton in most SURFIN/PvSTP proteins. To test this, in vitro binding experiments were performed by using other WRDs from SURFIN_4.2_ and PvSTP2 proteins with IOVs. In almost all cases, direct SURFIN/PvSTP WRD binding was observed with *K*_d_ values ranging from 0.26 to 0.68 µM. These modest binding values are of similar magnitude to the affinities of those observed in other *Plasmodium* proteins that are associated with RBC membrane skeleton, such as Pf332 (*K*_d_ = 0.40 µM) [7, 12, 29].

The potential binding between WRD2 of SURFIN_4.2_ and actin (an RBC membrane component) is implied from several clues. A previous report identified a fragment of 260 residues within the WRD of Pf332, which can bind to F-actin in a specific and saturable manner [12]. Pairwise sequence alignment revealed high sequence similarity between Pf332 and SURFIN/PvSTP proteins in this region [40], which makes actin the strongest candidate that interact with WRD2 of SURFIN_4.2_. This study tested the direct interaction between WRD2 of SURFIN_4.2_ and F-actin, which indeed confirmed such an interaction, albeit the interaction was relatively weak (*K*_d_ = 5.16 μM), and this interaction was abolished by further truncation of the recombinant protein. This value was more than eight folds higher than that detected for Pf332 in the previous study (*K*_d_ = 0.6 μM) [12] and other known parasite-host interacting proteins, including PfCor-N (*K*_d_ = 0.96 μM) and PfAldolase (*K*_d_ = 0.37 μM) [32, 41]. It is noteworthy that the *K*_d_ values of all examined WRDs of SURFIN_4.2_ and PvSTP2 with IOVs are similar and significantly higher than that of WRD of Pf332 and IOVs. Thus, the *K*_d_ value for the WRD2 of SURFIN_4.2_–F-actin interaction likely reflects the nature of the WRD2 of SURFIN_4.2_. It is tempting to speculate that the weak affinity of *P. falciparum* SURFIN WRD may be the driving force of the multiplication of this domain in the SURFIN family proteins in *P. falciparum*, which need to compete with other *P. falciparum*-specific proteins that also interact with actin and/or spectrin, such as PfEMP1, Pf332, and PfEMP3. However, it is also possible that the adjacent residues of the examined WRDs could enhance protein–protein interaction with RBC membrane skeleton, as has been reported for MESA interaction with protein 4.1 [42]. These possibilities need to be evaluated in the future. Interestingly, this study also identified interactions between the CRD of SURFIN_4.2_ and F-actin. However, this interaction may not be physiological since CRD and host F-actin are predicted to be located in different cellular compartments.

Another major component of the RBC membrane skeleton is spectrin, which is a flexible rod-like protein that predominantly exists as an α_2_β_2_ tetramer, and interacts with actin, protein 4.1, and ankyrin to form a network [43]. Several trafficked proteins to the RBC membrane interact with spectrin. Among them, RESA binds to the β chain of spectrin with a 108 amino acid fragment (residues 663–770), and stabilizes the spectrin tetramer and enhances resistance of the iRBCs to both mechanical and thermal degradation [29]. The spectrin-binding domain in KAHRP has been localized to a 72-residue region (residues 370–441), which is critical in membrane localization of KAHRP [24]. In the current study, the WRD2 of SURFIN_4.2_ also was found to interact with spectrin with the *K*_d_ value of 0.51 μM, which is comparable to that of RESA (0.88 μM) and PfEMP3 (0.38 μM) [29, 31]. Since no conserved motifs have been identified between SURFIN/PvSTP WRD and these previously identified spectrin-binding parasite proteins, it is possible that binding sites of spectrin for these parasite proteins might be different. The binding motifs in both spectrin and WRD2 of SURFIN_4.2_, and the functional role of this binding to spectrin remain to be further evaluated. Interestingly, the data presented here showed that the WRD2 of SURFIN_4.2_ was able to interact with both actin and spectrin. This is not an exception; a 14-residue fragment of PfEMP3 also has dual binding abilities to both spectrin and actin [7]. This dual binding ability detected in SURFIN_4.2_ and PfEMP3 may increase the affinity of these proteins for the iRBC membrane skeleton.

## Conclusions

This study identified SURFIN/PvSTP as a novel RBC membrane skeleton-binding protein family. The WRD2 of SURFIN_4.2_ was capable of interacting with both RBC membrane skeleton proteins actin and spectrin. These results imply that the WRDs of SURFIN/PvSTP proteins might be functionally conserved, and have evolved with the RBC skeleton interaction during protein trafficking process. Future studies will focus on pinpointing the key region(s) of the WRD2 of SURFIN_4.2_ which is involved in binding to actin and spectrin, as well as the role for these interactions. Understanding the parasite-host interaction mechanism, especially with regard to the surface expressed parasite antigens that are potentially involved in pathologic consequences, may facilitate the development of methods that interfere with these processes.

## Additional files

**Additional file 1: Table S1.** Primers for PCR amplification and plasmid construction.

**Additional file 2: Figure S1.** Sequence information of the His-tagged unrelated protein. The thioredoxin coding frame and protein sequences are shown in brown colour; The 6×His tag coding region and protein sequences are shown in blue colour; The multiple cloning sites are marked by underlines.

**Additional file 3: Figure S2.** Alignment of the tryptophan-rich domains (WRDs) of Pf332, SURFIN_4.2_/PvSTP2, and PfEMP1. Included in analysis are Pf332 WRD amino acid positions 5568-5825, WRDs of SURFIN_4.2_/PvSTP2 (WRD1 959-1201; WRD2 1349-1567; WRD3 1729-1990; PvSTP2 WRD 592-825), and PfEMP1 WRDs (PFD1005c WRD 1844-2181; PFD1015c WRD 1851-2193; PFL0030c WRD 2753-3056). Asterisks (“*”) indicate identical amino acids, while colon (“:”) and period (“.”) indicate conserved and semi-conserved amino acids, respectively. The identical/similar amino acids are shown in shading.

**Additional file 4: Figure S3.** Alignment of the amino acid sequences of the tryptophan-rich domains (WRDs) of SURFIN_4.1_ and SURFIN_4.2_ using the MUSCLE program. The remaining variable regions are shown in italic. The putative transmembrane domains (SURFIN_4.1_, amino acids 774-793; SURFIN_4.2_, amino acids 740-762) are shown in grey colour. The first and second WRDs (SURFIN_4.1_: WRD1 positions 908-1141, WRD2 1287-1476; SURFIN_4.2_: WRD1 959-1201, WRD2 1349-1567) are shown in yellow colour, and the positionally conserved tryptophan residues are shown in bold and red colour. Asterisks (“*”) indicate identical amino acids while colon (“:”) and period (“.”) indicate conserved and semi-conserved amino acids, respectively. SURFIN_4.1_ (accession no. AB759920.1); SURFIN_4.2_ (PlasmoDB ID: PF3D7_0424400).

**Additional file 5: Figure S4.** Preparation of inside-out vesicles (IOVs) from normal human RBCs. (**a**) Coomassie Brilliant Blue staining of separated protein present in the IOVs. (**b**) Western blot analysis of IOVs. IOVs were prepared and separated by SDS-PAGE, transferred to PVDF membrane, then detected with anti-actin or anti-spectrin antibodies. Recombinant actin was loaded as a positive control. Arrows indicate the spectrin dimer, protein 4.1 and actin.

**Additional file 6: Figure S5.** Scatchard analyses of the ELISA-based IOV binding assay of WRDs of SURFIN_4.2_ and PvSTP2. (**a**) Interactions between SURFIN_4.2_^WRD1^ and IOV. (**b**) Interactions between SURFIN_4.2_^WRD3^ and IOV. (**c**) Interactions between PvSTP2^WRD^ and IOV. (**d**) Interactions between Pf332^WRD^ and IOV.

**Additional file 7: Figure S6.** Binding of the His-tagged recombinant SURFIN_4.2_^WRD2−1^ and SURFIN_4.2_^CRD^ to F-actin. (**a**) The supernatant and (**b**) pellet fractions of SURFIN_4.2_^WRD2−1^-His (SURFIN_4.2_^WRD2−1^, WRD2-1 1349-1499, calculated MW, 38.0 kDa), SURFIN_4.2_^CRD^-His (SURFIN_4.2_^CRD^, CRD 1-197, calculated MW, 42.4 kDa), and His-tag control protein after F-actin co-sedimentation assay were analysed by Western blot with anti-His tag or anti-actin antibodies. Arrows indicate recombinant protein bands with the expected sizes.

**Additional file 8: Figure S7.** Preparation of the KAHRP spectrin-binding fragment. (**a**) Coomassie Brilliant Blue staining of KAHRP spectrin-binding fragment. (**b**) Western blot analysis of KAHRP spectrin-binding fragment 320-451. Arrows indicate His-tagged recombinant protein, KAHRP^320−451^-His.

## Abbreviations

iRBC: infected red blood cells
SURFIN: surface-associated interspersed protein
WRD: tryptophan-rich domain
CRD: cysteine-rich domain
MCs: Maurer’s clefts
PvSTPs: *Plasmodium vivax* subtelomeric transmembrane proteins
IOVs: inside-out vesicles
PVM: parasitophorous vacuole membrane
ELISA: enzyme-linked immunosorbent assay
PEXEL: *Plasmodium* exported element
PNEPs: PEXEL-negative proteins
VSAs: variant surface antigens

## References

[1] WHO (2015). World Malaria Report.

[2] McHugh E, Batinovic S, Hanssen E, McMillan PJ, Kenny S, Griffin MD (2015). A repeat sequence domain of the ring-exported protein-1 of *Plasmodium falciparum* controls export machinery architecture and virulence protein trafficking. Mol Microbiol.

[3] Dixon MW, Kenny S, McMillan PJ, Hanssen E, Trenholme KR, Gardiner DL (2011). Genetic ablation of a Maurer’s cleft protein prevents assembly of the *Plasmodium falciparum* virulence complex. Mol Microbiol.

[4] Oh SS, Voigt S, Fisher D, Yi SJ, LeRoy PJ, Derick LH (2000). *Plasmodium falciparum* erythrocyte membrane protein 1 is anchored to the actin-spectrin junction and knob-associated histidine-rich protein in the erythrocyte skeleton. Mol Biochem Parasitol.

[5] Mbengue A, Vialla E, Berry L, Fall G, Audiger N, Demettre-Verceil E (2015). New export pathway in *Plasmodium falciparum*-infected erythrocytes: role of the parasite group ii chaperonin. PfTRiC. Traffic..

[6] Alexandre JS, Yahata K, Kawai S, Torii M, Kaneko O (2011). PEXEL-independent trafficking of *Plasmodium falciparum* SURFIN_4.2_ to the parasite-infected red blood cell and Maurer’s clefts. Parasitol Int.

[7] Waller KL, Stubberfield LM, Dubljevic V, Nunomura W, An X, Mason AJ (2007). Interactions of *Plasmodium falciparum* erythrocyte membrane protein 3 with the red blood cell membrane skeleton. Biochim Biophys Acta.

[8] Chan JA, Howell KB, Langer C, Maier AG, Hasang W, Rogerson SJ (2016). A single point in protein trafficking by *Plasmodium falciparum* determines the expression of major antigens on the surface of infected erythrocytes targeted by human antibodies. Cell Mol Life Sci.

[9] Rug M, Prescott SW, Fernandez KM, Cooke BM, Cowman AF (2006). The role of KAHRP domains in knob formation and cytoadherence of *P. falciparum*-infected human erythrocytes. Blood.

[10] Kats LM, Proellocks NI, Buckingham DW, Blanc L, Hale J, Guo X (2015). Interactions between *Plasmodium falciparum* skeleton-binding protein 1 and the membrane skeleton of malaria-infected red blood cells. Biochim Biophys Acta.

[11] Da Silva E, Foley M, Dluzewski AR, Murray LJ, Anders RF, Tilley L (1994). The *Plasmodium falciparum* protein RESA interacts with the erythrocyte cytoskeleton and modifies erythrocyte thermal stability. Mol Biochem Parasitol.

[12] Waller KL, Stubberfield LM, Dubljevic V, Buckingham DW, Mohandas N, Coppel RL (2010). Interaction of the exported malaria protein Pf332 with the red blood cell membrane skeleton. Biochim Biophys Acta.

[13] Black CG, Proellocks NI, Kats LM, Cooke BM, Mohandas N, Coppel RL (2008). In vivo studies support the role of trafficking and cytoskeletal-binding motifs in the interaction of MESA with the membrane skeleton of *Plasmodium falciparum*-infected red blood cells. Mol Biochem Parasitol.

[14] Winter G, Kawai S, Haeggstrom M, Kaneko O, von Euler A, Kawazu S (2005). SURFIN is a polymorphic antigen expressed on *Plasmodium falciparum* merozoites and infected erythrocytes. J Exp Med.

[15] Sungkapong T, Culleton R, Yahata K, Tachibana M, Ruengveerayuth R, Udomsangpetch R (2011). Humoral immune responses to *Plasmodium vivax* subtelomeric transmembrane proteins in Thailand. Southeast Asian J Trop Med Public Health.

[16] Chan JA, Fowkes FJ, Beeson JG (2014). Surface antigens of *Plasmodium falciparum*-infected erythrocytes as immune targets and malaria vaccine candidates. Cell Mol Life Sci.

[17] Ansari FA, Kumar N, Bala Subramanyam M, Gnanamani M, Ramachandran S (2008). MAAP: malarial adhesins and adhesin-like proteins predictor. Proteins.

[18] Zhu X, Yahata K, Alexandre JS, Tsuboi T, Kaneko O (2013). The N-terminal segment of *Plasmodium falciparum* SURFIN_4.1_ is required for its trafficking to the red blood cell cytosol through the endoplasmic reticulum. Parasitol Int.

[19] Kagaya W, Miyazaki S, Yahata K, Ohta N, Kaneko O (2015). The cytoplasmic region of *Plasmodium falciparum* SURFIN_4.2_ is required for transport from Maurer’s clefts to the red blood cell surface. Trop Med Health.

[20] Waller KL, Cooke BM, Nunomura W, Mohandas N, Coppel RL (1999). Mapping the binding domains involved in the interaction between the *Plasmodium falciparum* knob-associated histidine-rich protein (KAHRP) and the cytoadherence ligand *P. falciparum* erythrocyte membrane protein 1 (PfEMP1). J Biol Chem.

[21] Ganguly AK, Ranjan P, Kumar A, Bhavesh NS (2015). Dynamic association of PfEMP1 and KAHRP in knobs mediates cytoadherence during *Plasmodium* invasion. Sci Rep..

[22] Kunkel TA (1985). Rapid and efficient site-specific mutagenesis without phenotypic selection. Proc Natl Acad Sci USA.

[23] Tonkin CJ, van Dooren GG, Spurck TP, Struck NS, Good RT, Handman E (2004). Localization of organellar proteins in *Plasmodium falciparum* using a novel set of transfection vectors and a new immunofluorescence fixation method. Mol Biochem Parasitol.

[24] Pei X, An X, Guo X, Tarnawski M, Coppel R, Mohandas N (2005). Structural and functional studies of interaction between *Plasmodium falciparum* knob-associated histidine-rich protein (KAHRP) and erythrocyte spectrin. J Biol Chem.

[25] Artimo P, Jonnalagedda M, Arnold K, Baratin D, Csardi G, de Castro E (2012). ExPASy: SIB bioinformatics resource portal. Nucleic Acids Res.

[26] Spycher C, Klonis N, Spielmann T, Kump E, Steiger S, Tilley L (2003). MAHRP-1, a novel *Plasmodium falciparum* histidine-rich protein, binds ferriprotoporphyrin IX and localizes to the Maurer’s clefts. J Biol Chem.

[27] de Koning-Ward TF, Dixon MW, Tilley L, Gilson PR (2016). *Plasmodium* species: master renovators of their host cells. Nat Rev Microbiol.

[28] Bachmann A, Scholz JA, Janssen M, Klinkert MQ, Tannich E, Bruchhaus I (2015). A comparative study of the localization and membrane topology of members of the RIFIN, STEVOR and PfMC-2TM protein families in *Plasmodium falciparum*-infected erythrocytes. Malar J.

[29] Pei X, Guo X, Coppel R, Bhattacharjee S, Haldar K, Gratzer W (2007). The ring-infected erythrocyte surface antigen (RESA) of *Plasmodium falciparum* stabilizes spectrin tetramers and suppresses further invasion. Blood.

[30] Waller KL, Nunomura W, An X, Cooke BM, Mohandas N, Coppel RL (2003). Mature parasite-infected erythrocyte surface antigen (MESA) of *Plasmodium falciparum* binds to the 30-kDa domain of protein 4.1 in malaria-infected red blood cells. Blood.

[31] Pei X, Guo X, Coppel R, Mohandas N, An X (2007). *Plasmodium falciparum* erythrocyte membrane protein 3 (PfEMP3) destabilizes erythrocyte membrane skeleton. J Biol Chem.

[32] Olshina MA, Angrisano F, Marapana DS, Riglar DT, Bane K, Wong W (2015). *Plasmodium falciparum* coronin organizes arrays of parallel actin filaments potentially guiding directional motility in invasive malaria parasites. Malar J.

[33] Spycher C, Rug M, Pachlatko E, Hanssen E, Ferguson D, Cowman AF (2008). The Maurer’s cleft protein MAHRP1 is essential for trafficking of PfEMP1 to the surface of *Plasmodium falciparum*-infected erythrocytes. Mol Microbiol.

[34] Kriek N, Tilley L, Horrocks P, Pinches R, Elford BC, Ferguson DJ (2003). Characterization of the pathway for transport of the cytoadherence-mediating protein, PfEMP1, to the host cell surface in malaria parasite-infected erythrocytes. Mol Microbiol.

[35] Saridaki T, Frohlich KS, Braun-Breton C, Lanzer M (2009). Export of PfSBP1 to the *Plasmodium falciparum* Maurer’s clefts. Traffic.

[36] Haase S, Herrmann S, Gruring C, Heiber A, Jansen PW, Langer C (2009). Sequence requirements for the export of the *Plasmodium falciparum* Maurer’s clefts protein REX2. Mol Microbiol.

[37] Dixon MW, Hawthorne PL, Spielmann T, Anderson KL, Trenholme KR, Gardiner DL (2008). Targeting of the ring exported protein 1 to the Maurer’s clefts is mediated by a two-phase process. Traffic.

[38] Cunningham D, Lawton J, Jarra W, Preiser P, Langhorne J (2010). The pir multigene family of *Plasmodium*: antigenic variation and beyond. Mol Biochem Parasitol.

[39] Ansari HR, Templeton TJ, Subudhi AK, Ramaprasad A, Tang J, Lu F (2016). Genome-scale comparison of expanded gene families in *Plasmodium ovale wallikeri* and *Plasmodium ovale curtisi* with *Plasmodium malariae* and with other *Plasmodium* species. Int J Parasitol.

[40] Frech C, Chen N (2013). Variant surface antigens of malaria parasites: functional and evolutionary insights from comparative gene family classification and analysis. BMC Genomics.

[41] Diaz SA, Martin SR, Grainger M, Howell SA, Green JL, Holder AA (2014). *Plasmodium falciparum* aldolase and the C-terminal cytoplasmic domain of certain apical organellar proteins promote actin polymerization. Mol Biochem Parasitol.

[42] Kun JF, Waller KL, Coppel RL (1999). *Plasmodium falciparum*: structural and functional domains of the mature-parasite-infected erythrocyte surface antigen. Exp Parasitol.

[43] Bennett V, Gilligan DM (1993). The spectrin-based membrane skeleton and micron-scale organization of the plasma membrane. Annu Rev Cell Biol.

