# Supplementary material for: Tryptophan-rich domains of Plasmodium falciparum SURFIN4.2 and Plasmodium vivax PvSTP2 interact with membrane skeleton of red blood cell
Source: Malar J. 2017 Mar 20;16:121. doi: 10.1186/s12936-017-1772-5 (PMC5359885; doi:10.1186/s12936-017-1772-5)
Supplement: Supplementary file 1 — Additional file 1: Table S1. Primers for PCR amplification and plasmid construction. [file 12936_2017_1772_MOESM1_ESM.docx]

**S1 Table. Primers for PCR fragments amplification and plasmids construction**

| Plasmids | Primer | Sequence (5’ to 3’) |
| --- | --- | --- |
| pENT12-SURFIN_4.1_^2Myc-N-T-Cyt^ | pENNTC-Myc.F | ctggaacagaagttaataagtgaggaagacCATTTTGTAGTTGAATTGG |
|  | pENNTC-Myc.R | atcctcttctgagatgagtttttgttcgggCATTGTTTATTAGCCTGC |
| pENT12-SURFIN_4.1_^2Myc-N-T-Cyt-StuI^ | pEN12Myc-StuI-F | *cct*tttCAGCTTTCTTGTACAAAGTTGGC |
|  | pEN12Myc-StuI-R | *cct*tttGGTCCATCATACCTTCTTCGTC |
| pENT12-SURFIN_4.1_^2Myc-N-T-Cyt-4.2WRD2^ | SURF42-WR_2_.StuIF | ggGAAAAAAAAGTAAAATGGAAAACTATCATTG |
|  | SURF42-WR_2_.StuIR | ggccTGTATCAACAAATGTTTGTTTATTCCTATAAC |
| SURFIN_4.2_^WRD1^ | SURF42WR_1_.B1F | ggggACAAGTTTGTACAAAAAAGCAGGCTTAAAAAAGGAGAAATGGATGTGGAAAAC |
|  | SURF42WR_1_.B2R | ggggACCACTTTGTACAAGAAAGCTGGGTAATTGTCTAATAATAAATTCTTAATATG |
| SURFIN_4.2_^WRD2^ | SURF42WR_2_.B1F | ggggACAAGTTTGTACAAAAAAGCAGGCTGAAAAAAAAGTAAAATGGAAAACTATCATTG |
|  | SURF42WR_2_.B2R | ggggACCACTTTGTACAAGAAAGCTGGGTTGTATCAACAAATGTTTGTTTATTCCTATAAC |
| SURFIN_4.2_^WRD2-1^ | SURF42WR2-1.B1F | ggggACAAGTTTGTACAAAAAAGCAGGCT*tgaag*GAAAAAAAAGTAAAATGG |
|  | SURF42WR2-1.B2R | ggggACCACTTTGTACAAGAAAGCTGGGT*a*CCACTCATCAATATGATATAAATG |
| SURFIN_4.2_^WRD3^ | SURF42WR_3_.B1F | ggggACAAGTTTGTACAAAAAAGCAGGCT*atgaa*AATAATCATTCCTATATTAAAAGG |
|  | SURF42WR_3_.B2R | ggggACCACTTTGTACAAGAAAGCTGGGTaTGAATTTAAAAATATTTCTTTATTATATC |
| SURFIN_4.2_^CRD^ | SURF42CRD.B1F | ggggACAAGTTTGTACAAAAAAGCAGGCT*ataat*ATGCTTTTTGTTGTTGAGCTCGAC |
|  | SURF42CRD.B2R | ggggACCACTTTGTACAAGAAAGCTGGGT*t*ATCCTCACTTGATATATTAAGG |
| PvSTP2^WRD^ | PvSTP2WR.B1F | ggggACAAGTTTGTACAAAAAAGCAGGCT*caaag*AAAAAAAAAAGTGGCTCAAAAACG |
|  | PvSTP2WR.B2R | ggggACCACTTTGTACAAGAAAGCTGGGT*c*GTCATCCAAGTACAACTCCCTATTTTGC |
| Pf332^WRD^ | Pf332WR.BamHI | cg*ggatcc*AATGATACTGTAATGGTTATAAAAATC |
|  | Pf332WR.NotI | ttttcctttt*gcggccgc*AAGTTCATCGTACTTAAATTGATC |
| KAHRP^370-441^ | KAHRPsp_attB1F | ggggACAAGTTTGTACAAAAAAGCAGGCT*cc*GTCCCACCCCATGGTGCAGGC |
|  | KAHRPsp_attB2R | ggggACCACTTTGTACAAGAAAGCTGGGT*g*GCTGCATGGTTTTTTTCCATTATGC |
