# Supplementary figures and images for: Tryptophan-rich domains of Plasmodium falciparum SURFIN4.2 and Plasmodium vivax PvSTP2 interact with membrane skeleton of red blood cell
Source: Malar J. 2017 Mar 20;16:121. doi: 10.1186/s12936-017-1772-5 (PMC5359885; doi:10.1186/s12936-017-1772-5)

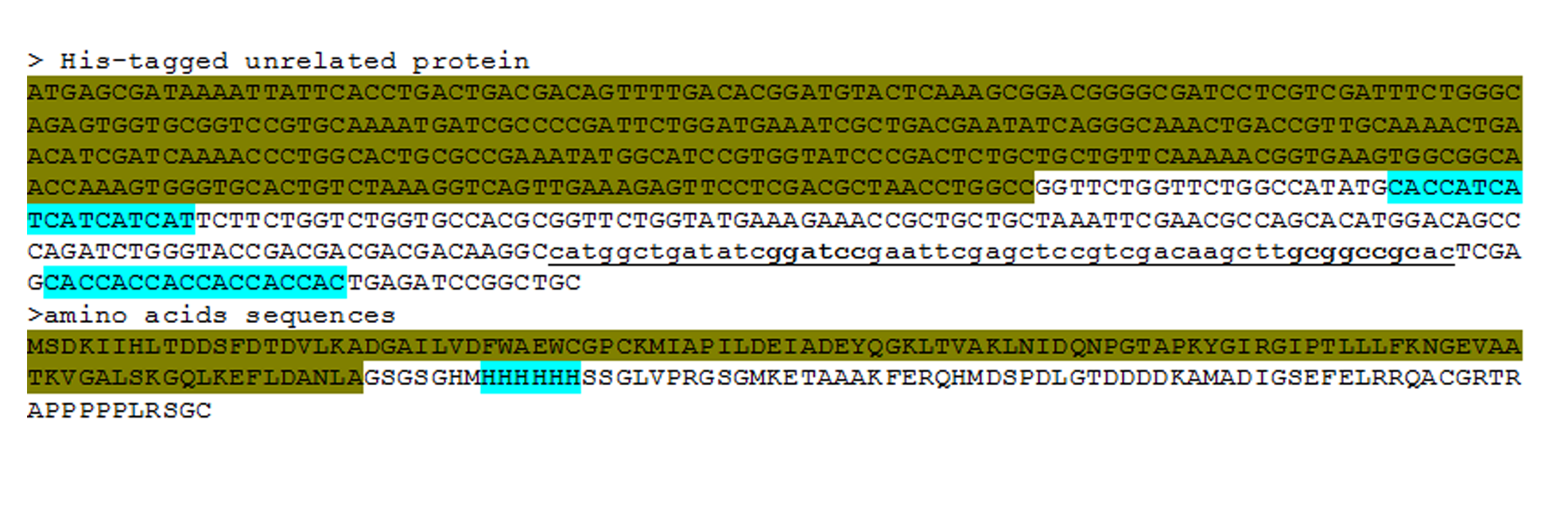

Supplement: Supplementary file 2 — Additional file 2: Figure S1. Sequence information of the His-tagged unrelated protein. The thioredoxin coding frame and protein sequences are shown in brown colour; The 6×His tag coding region and protein sequences are shown in blue colour; The multiple cloning sites are marked by underlines. [file 12936_2017_1772_MOESM2_ESM.tif]

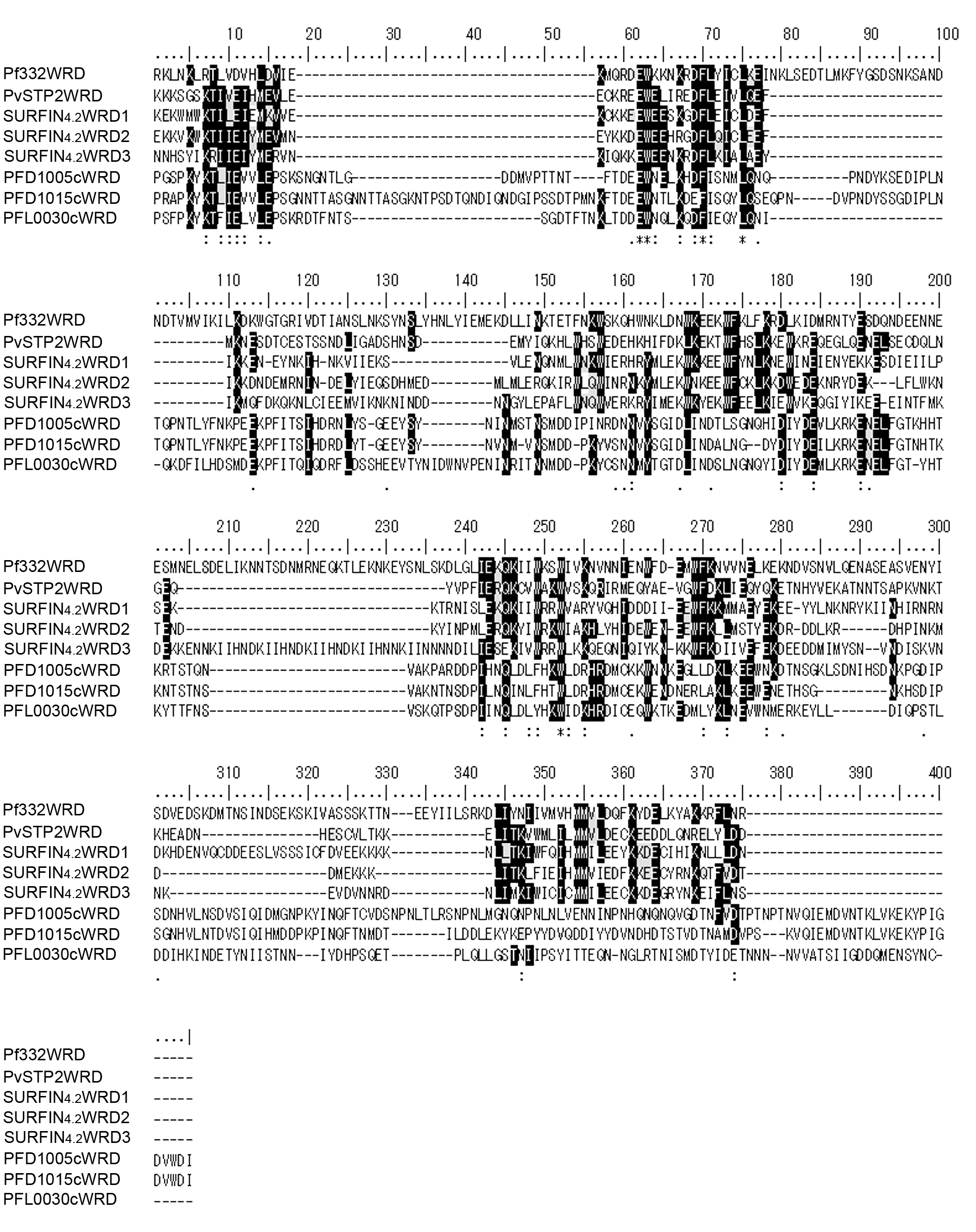

Supplement: Supplementary file 3 — Additional file 3: Figure S2. Alignment of the tryptophan-rich domains (WRDs) of Pf332, SURFIN4.2/PvSTP2, and PfEMP1. Included in analysis are Pf332 WRD amino acid positions 5568-5825, WRDs of SURFIN4.2/PvSTP2 (WRD1 959-1201; WRD2 1349-1567; WRD3 1729-1990; PvSTP2 WRD 592-825), and PfEMP1 WRDs (PFD1005c WRD 1844-2181; PFD1015c WRD 1851-2193; PFL0030c WRD 2753-3056). Asterisks (“*”) indicate identical amino acids, while colon (“:”) and period (“.”) indicate conserved and semi-conserved amino acids, respectively. The identical/similar amino acids are shown in shading. [file 12936_2017_1772_MOESM3_ESM.tif]

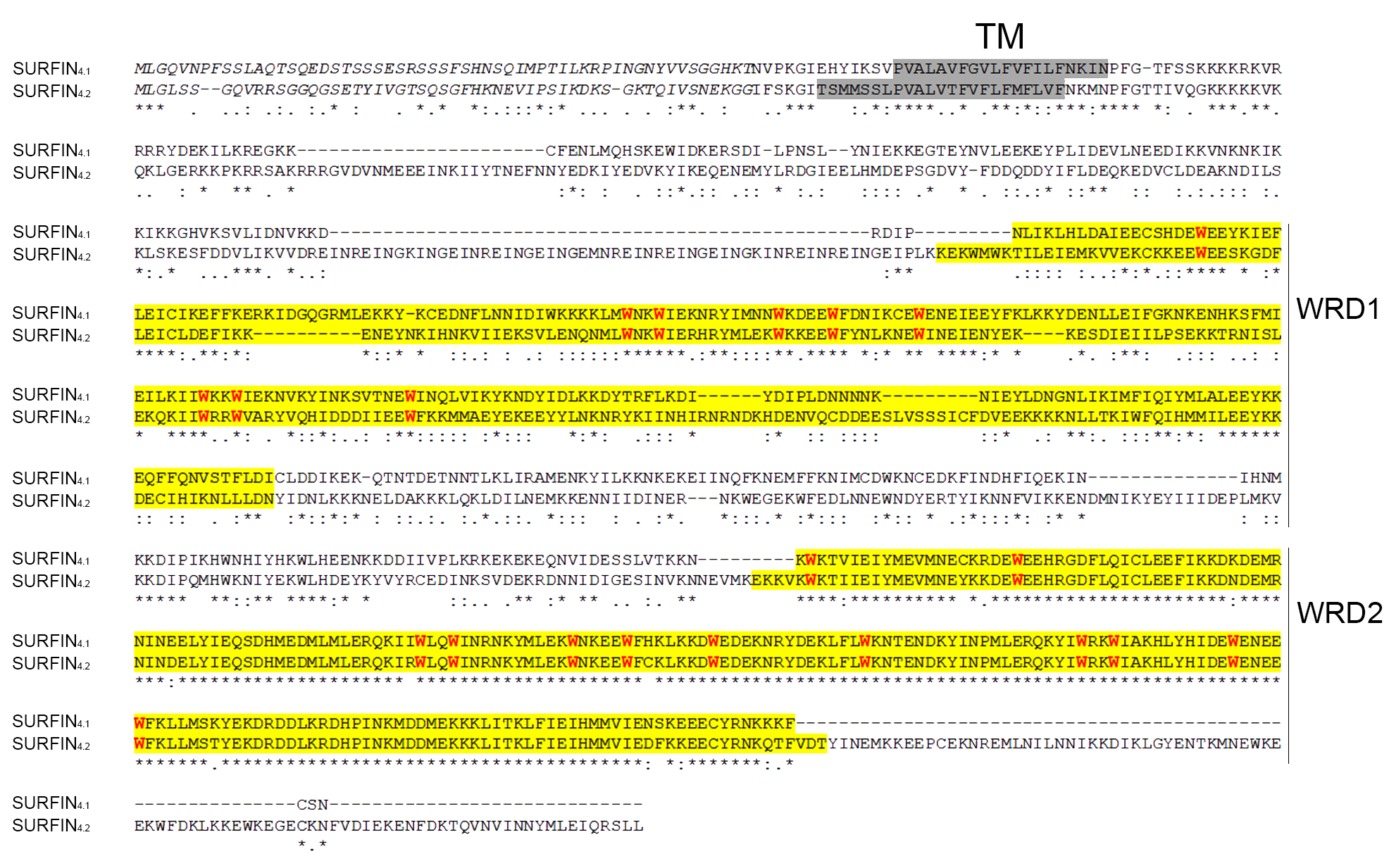

Supplement: Supplementary file 4 — Additional file 4: Figure S3. Alignment of the amino acid sequences of the tryptophan-rich domains (WRDs) of SURFIN4.1 and SURFIN4.2 using the MUSCLE program. The remaining variable regions are shown in italic. The putative transmembrane domains (SURFIN4.1, amino acids 774-793; SURFIN4.2, amino acids 740-762) are shown in grey colour. The first and second WRDs (SURFIN4.1: WRD1 positions 908-1141, WRD2 1287-1476; SURFIN4.2: WRD1 959-1201, WRD2 1349-1567) are shown in yellow colour, and the positionally conserved tryptophan residues are shown in bold and red colour. Asterisks (“*”) indicate identical amino acids while colon (“:”) and period (“.”) indicate conserved and semi-conserved amino acids, respectively. SURFIN4.1 (accession no. AB759920.1); SURFIN4.2 (PlasmoDB ID: PF3D7_0424400). [file 12936_2017_1772_MOESM4_ESM.tif]

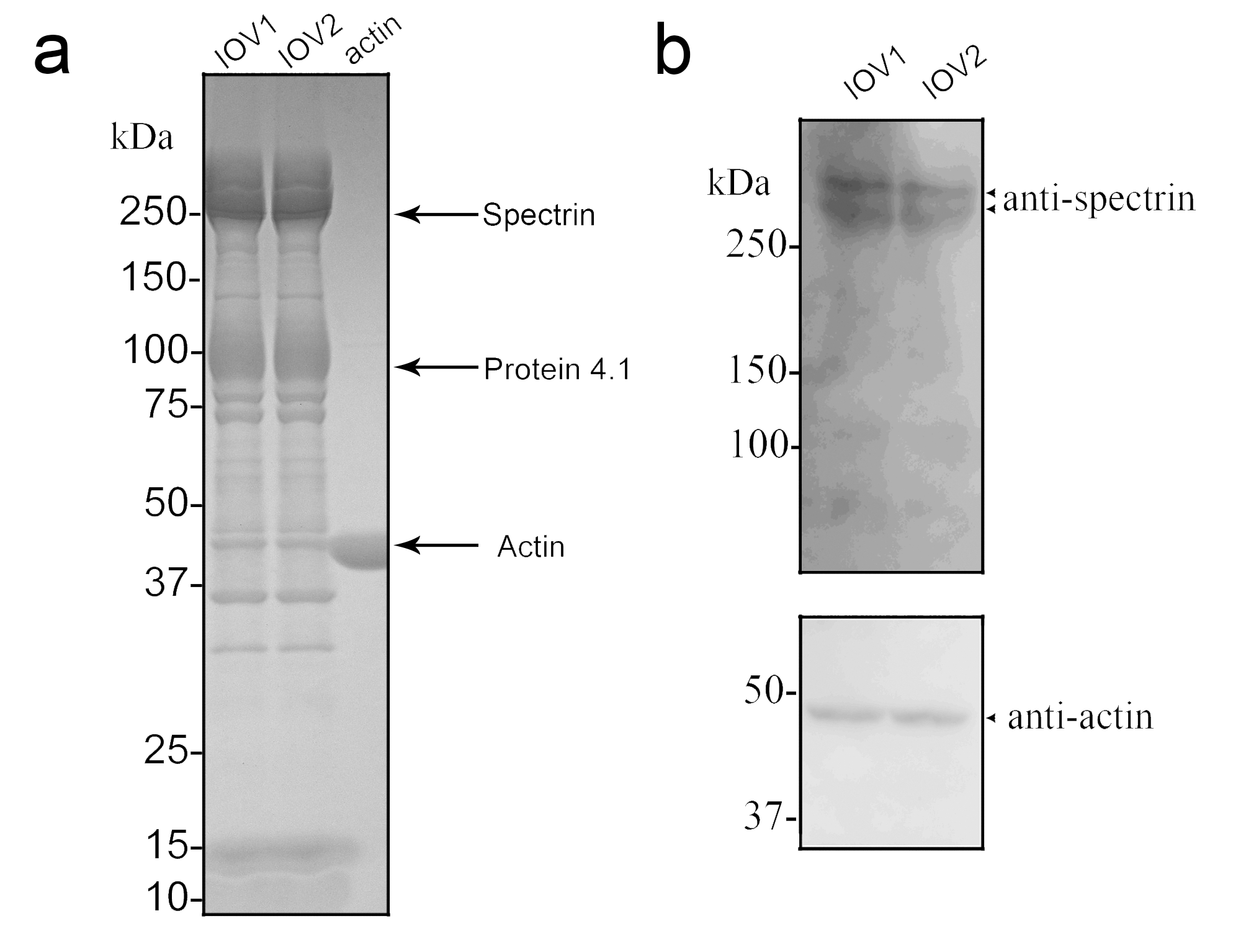

Supplement: Supplementary file 5 — Additional file 5: Figure S4. Preparation of inside-out vesicles (IOVs) from normal human RBCs. (a) Coomassie Brilliant Blue staining of separated protein present in the IOVs. (b) Western blot analysis of IOVs. IOVs were prepared and separated by SDS-PAGE, transferred to PVDF membrane, then detected with anti-actin or anti-spectrin antibodies. Recombinant actin was loaded as a positive control. Arrows indicate the spectrin dimer, protein 4.1 and actin. [file 12936_2017_1772_MOESM5_ESM.tif]

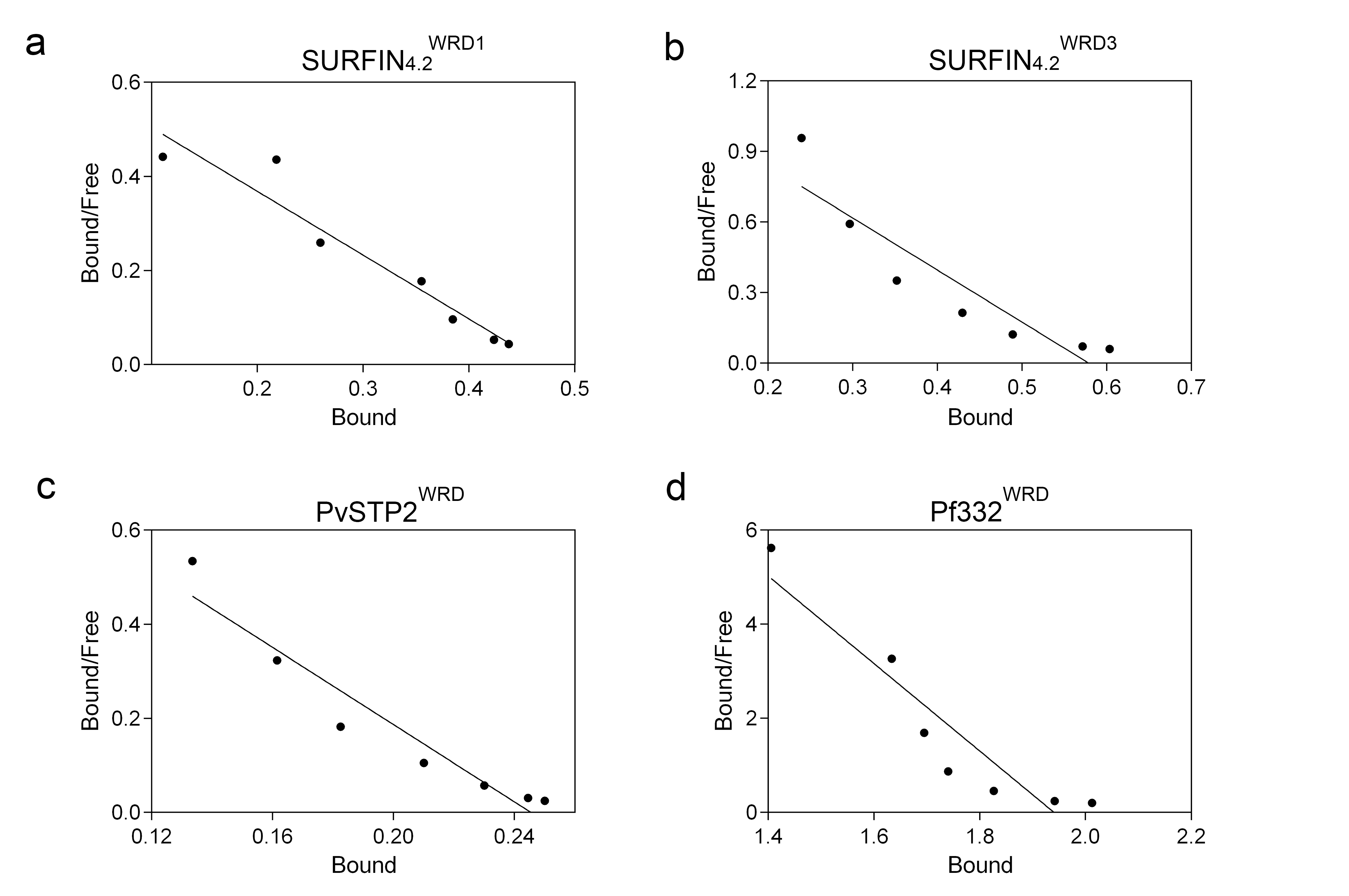

Supplement: Supplementary file 6 — Additional file 6: Figure S5. Scatchard analyses of the ELISA-based IOV binding assay of WRDs of SURFIN4.2 and PvSTP2. (a) Interactions between SURFIN4.2WRD1 and IOV. (b) Interactions between SURFIN4.2WRD3 and IOV. (c) Interactions between PvSTP2WRD and IOV. (d) Interactions between Pf332WRD and IOV. [file 12936_2017_1772_MOESM6_ESM.tif]

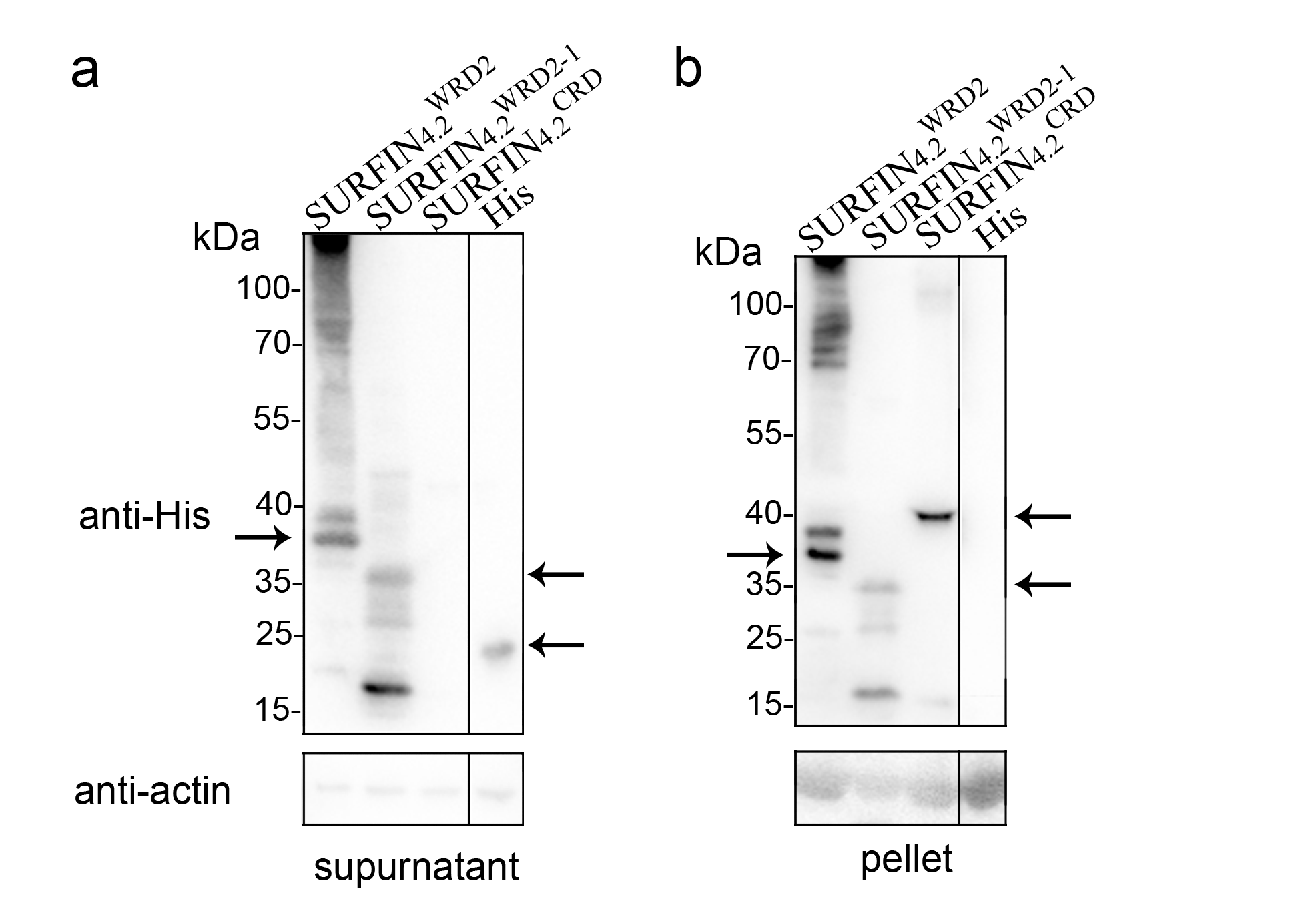

Supplement: Supplementary file 7 — Additional file 7: Figure S6. Binding of the His-tagged recombinant SURFIN4.2WRD2−1 and SURFIN4.2CRD to F-actin. (a) The supernatant and (b) pellet fractions of SURFIN4.2WRD2−1-His (SURFIN4.2WRD2−1, WRD2-1 1349-1499, calculated MW, 38.0 kDa), SURFIN4.2CRD-His (SURFIN4.2CRD, CRD 1-197, calculated MW, 42.4 kDa), and His-tag control protein after F-actin co-sedimentation assay were analysed by Western blot with anti-His tag or anti-actin antibodies. Arrows indicate recombinant protein bands with the expected sizes. [file 12936_2017_1772_MOESM7_ESM.tif]

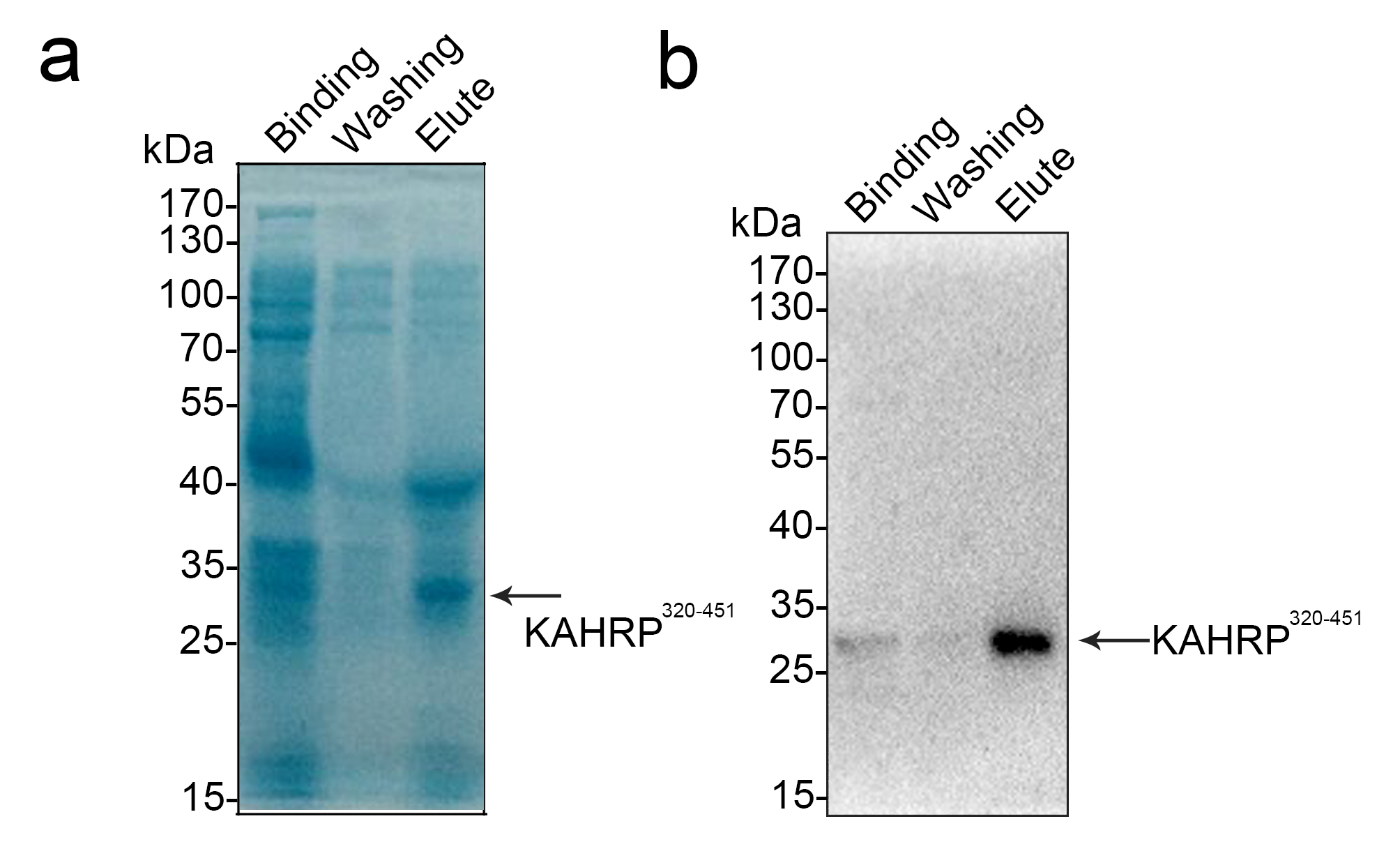

Supplement: Supplementary file 8 — Additional file 8: Figure S7. Preparation of the KAHRP spectrin-binding fragment. (a) Coomassie Brilliant Blue staining of KAHRP spectrin-binding fragment. (b) Western blot analysis of KAHRP spectrin-binding fragment 320-451. Arrows indicate His-tagged recombinant protein, KAHRP320−451-His. [file 12936_2017_1772_MOESM8_ESM.tif]
